# Functional Cerebral Specialization and Decision Making in the Iowa Gambling Task: A Single-Case Study of Left-Hemispheric Atrophy and Hemispherotomy

**DOI:** 10.3389/fpsyg.2020.00725

**Published:** 2020-04-21

**Authors:** Varsha Singh, Kapil Chaudhary, S. Senthil Kumaran, Sarat Chandra, Manjari Tripathi

**Affiliations:** ^1^Psychology, Humanities and Social Sciences, Indian Institute of Technology Delhi, New Delhi, India; ^2^Department of Neurology, Neuroscience Centre, All India Institute of Medical Sciences, New Delhi, India; ^3^Department of Nuclear Magnetic Resonance, All India Institute of Medical Sciences, New Delhi, India; ^4^Department of Neurosurgery, Neuroscience Centre, All India Institute of Medical Sciences, New Delhi, India

**Keywords:** decision making, hemispheric specialization, Iowa Gambling Task, lateralized function, valence

## Abstract

The Iowa Gambling Task (IGT) is a decision-making task that preferentially involves the right prefrontal cortex (PFC). However, the performance of the task is driven by two attributes: intertemporal (long vs. short-term) and frequency-based processing of rewards-punishments, and differs over the two phases of uncertainty (early trials) and risk (later trials). Although intertemporal decision making involves the right PFC, the extent of hemispheric specialization in attribute and phase-specific decision making is unknown. Therefore, the current study assessed decision making in a patient with a uni-hemispheric disease, who underwent hemispherotomy surgery, comparing pre-surgical IGT performance (3 days prior to surgery) with post-surgical performance (1 month, and 12 months post-surgery). The patient’s pre- and post-surgical IGT performances were analyzed to examine changes in attribute and phase-specific decision making, including the widely reported deck B phenomenon. The results for the two attributes of deck selection at the pre- and post-surgical assessments suggested marked changes in the two IGT phases of risk and uncertainty. Pre-surgery, the patient made more intertemporally disadvantageous choices, and task-progression contributed to it; within 1 month of surgery, intertemporal disadvantageous deck choices were contingent on task progression, after 1 year, disadvantageous choices were independent of task progression. Intertemporal attribute alteration was unresponsive to uncertainty and risk phase. The effect of task progression on frequency attribute remained unchanged before and immediately after the surgery, and preference for infrequent decks was observed only after 1 year. Further, pre and post surgery alteration in frequency attribute was phase-specific: within 1 month of surgery, infrequent deck choices decreased in uncertainty and increased in risk, whereas the reverse was observed after 12 months. Deck B choice increase was in the uncertainty phase. Results are discussed in reference to valence-linked hemispheric specialization and its potential role in attribute and phase-specific IGT decision making.

## Background

The Iowa Gambling Task (IGT) was devised to account for decision making deficits in patients with focal damage in the ventromedial prefrontal cortex (PFC; Damasio, 1996). The PFC enables somatic markers to guide long-term decision making in the IGT (see for task description; Bechara et al., 2005). Over a period of time, the IGT has been considered as complex task such that usage of two types of decision making strategies have been observed: cognition-intensive processing of long term pay-offs (over a period of trials), intertemporal and emotion-based, and automatic, frequency-based decision making (choices made based on the frequency of rewards and punishments) (Stocco et al., 2009). These two attributes contribute to the heterogeneity observed in the IGT (Singh and Khan, 2009). Further, there are different phases in the 100 trials of the task that assess different constructs; the initial 50 trials, where punishments get introduced, assess decision making under uncertainty (unknown payoffs/outcomes), whereas the subsequent trials (after 50) evaluate decision making under risk (known payoffs/outcomes) (Brand et al., 2007; Bagneux et al., 2013; Singh et al., 2020). Some researchers have observed the intertemporal attribute decision making to differ between the first half trials and the last half trials (e.g., Rocha et al., 2011; Steingroever et al., 2013). Further, the first and the last phase vary in cognitive demands, the earlier uncertainty phase has the lesser demands (Bagneux et al., 2013), whereas the last risk phase is more demanding of cognitive resources (Brand et al., 2007). Therefore, there is a possibility of attribute, and phase-specificity in the IGT decision making; however, most of the IGT studies focus solely on the intertemporal attribute, and consider all trials of the IGT to be homogenous in terms of the cognitive demands.

This far, studies of laterality in the IGT have offered insight in intertemporal decision making and how it progresses across the 100 task trials (Bolla et al., 2003; Clark et al., 2003; Fukui et al., 2005; Li et al., 2010). For instance, studies have suggested that right PFC injury results in poor intertemporal decision making in the IGT (Clark et al., 2003), whereas lesions in the left hemisphere do not impair intertemporal task performance (Tranel et al., 2002), the extent of hemispheric laterality involved in attribute and phase-specificity in IGT decision making remains unclear. Further, the right-PFC link to the IGT intertemporal decision making shows sex-specificity: male decision making is impaired by the right ventromedial PFC damage, while left-hemispheric damage impairs female intertemporal decision making in the IGT (Tranel et al., 2005). Aligned with the contention of lesion laterality, male advantage in the task’s intertemporal decision making is attributed to greater right-hemispheric specialization in men (van den Bos et al., 2013). Frequency-based choice of infrequent punishment deck B is intertemporally disadvantageous (Lin et al., 2007), but this preference for infrequent punishment/losses is prominent in females (Overman and Pierce, 2013). Sex-differences are not limited to attribute-specificity but also show phase-specificity. For instance, the deck B choice among females was observed in the uncertainty phase (Okdie et al., 2016). One potential reason for the speculation of hemispheric role in attribute and phase-specificity in the IGT decision making might be that the processing of uncertainty shows lateralization (Goel et al., 2007; Marinsek et al., 2014). Additionally, uncertainty is linked with anticipatory anxiety or negative valence about the future emotion states (Grupe and Nitschke, 2013). Hemispheric lateralization linked to valence processing might contribute to sex differences in intertemporal attribute (Bolla et al., 2004; Singh, 2016), however, hemispheric role in attribute and phase-specificity in IGT decision-making remains poorly understood.

Functional cerebral specialization may influence IGT decision making in attribute, and phase-specific manner (i.e., uncertainty and risk), and this presents a need for understanding of hemispheric specialization in the IGT. However, examining hemispheric specialization while accounting for interhemispheric connectivity and exchange is a challenge that can be addressed by examining the effects of uni-hemispheric damage on the task across the disconnection of the two hemispheres (Gazzaniga, 2000). We herein present the case of a woman with Rasmussen encephalitis, a condition characterized by uni-hemispheric damage and the occurrence of epileptic seizures; in the present case, the patient exhibited left-hemispheric atrophy. Due to drug resistance, seizure-alleviation was attained with hemispherotomy (Chandra and Tripathi, 2015): a procedure that functionally dissociated the epileptogenic left hemisphere from the non-epileptogenic hemisphere.

Rare neurological drug-resistant epilepsy such as the Rasmussen Encephalitis entails uni-hemispheric atrophy, further; the treatment is dissociation of the epileptogenic hemisphere. Even though the deficit in the IGT cannot be attributed to solely to epilepsy-linked structural or functional atrophy (Delazer et al., 2010), uni-hemispheric atrophy and post-surgery changes offer insights about the hemispheric contribution to cognitive functions, for instance, uni-hemispheric epilepsy localized either to the right or to the left hemisphere did not differ in terms of the deficit observed in the intertemporal IGT decision making (Bonatti et al., 2009; Labudda et al., 2009; Delazer et al., 2010, 2011). This observation is reported in a recent meta analysis of the IGT epilepsy studies (Zhang et al., 2018), however, so far the studies have either analyzed intertemporal IGT decision making prior to the hemispheric dissociation (e.g., Labudda et al., 2009; Delazer et al., 2010) or after the surgery (e.g., Butman et al., 2007; Bonatti et al., 2009). To our knowledge, this is the first case study of a female patient with left hemispheric atrophy, whose IGT performance is analyzed for pre and post surgery changes within a short (1 month) and a long duration (12 months) of hemispheric dissociation. Others have used single case study of epilepsy for understanding emotion processing (e.g., Tamune et al., 2018). Therefore, a rare case of female patient of Rasmussen Encephalitis epilepsy with left hemispheric atrophy is used herewith for understanding how the two attributes (i.e., cognition-intensive and emotion-based attributes) in two phases differing in cognitive demands (i.e., uncertainty and risk) influence performance in a task that shows right-laterality-linked male advantage. It was expected that uni-hemispheric damage and a comparison of the patient’s performance after hemispherotomy enabled examination of the attribute and phase-specific IGT decision-making changes occurring within 1 month and 12 months of post-hemispheric disconnection. Further, we explored pre and post-surgery changes in valence processing (pictures and words of positive, negative and neutral valence) as a possible contributor to attribute and phase-specificity in the IGT.

## Case Report

A 26-year-old woman with late-onset, drug-resistant left-hemispheric Rasmussen encephalitis (onset age: 15 years; seizure duration: 12 years) presented with aphasia and progressive right hemiparesis. She was admitted with a 6-year history of progressive right hemiparesis, a 4-year history of intact comprehension with impaired speech production, and attendant progressive cognitive impairment. In preparation for the surgery, neuropsychological evaluation, brain imaging [magnetic resonance imaging (MRI), three-dimensional fluid-attenuated inversion recovery (3D FLAIR) imaging, functional MRI (fMRI)], and semiology tests were performed. We used pre-surgical evaluations to ascertain the following abnormalities: left-hemispheric atrophy, left-lateralized atrophy in areas commonly implicated in the IGT, and functional left-lateralization of language (motor speech production and word comprehension). The Ethics Committee at the medical institute approved the study protocol. Written informed consent for study-participation, and publication of this report was obtained from the participant and her caregiver.

The patient could not complete the routine pre-surgical neuropsychological evaluations (Mental State Exam: MSE). The auditory verbal functioning and visuospatial functioning tests were attempted but could not be completed on account of the patient’s seizures and speech difficulties. The summary of the neuropsychological evaluation suggested that the patient had impaired intellectual functioning and an absence of anxiety or depressive symptoms.

### Left-Hemisphere Atrophy and Left-Lateralized Atrophy in IGT Circuitry

**FIGURE 1:**
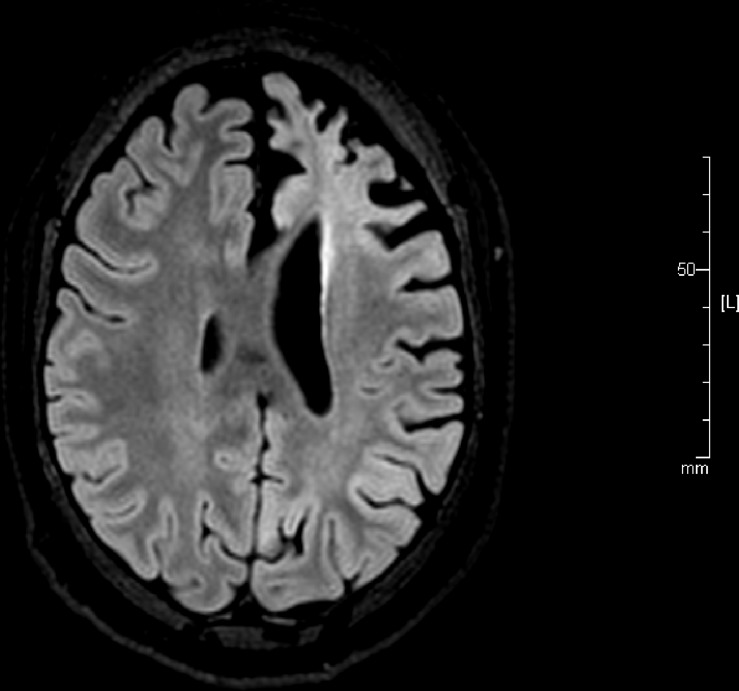
A reconstructed axial three-dimensional fluid-attenuated inversion recovery image obtained from the patient with left-hemispheric Rasmussen encephalitis before surgery shows atrophy in the left hemisphere indicated by ‘L’ (repetition time: 4800 ms, echo time: 270.9 ms, slice thickness: 1.0 mm, matrix size 512 × 512).

Assessing the state of the left hemisphere, the pre-surgical seizure semiology test findings were used to verify focal onset of seizures in the left hemisphere with clonic movements of the right limbs (upper and lower) and right facial twitching (video-EEG). Subsequent visual inspection of 3D FLAIR images (Figure 1) verified left-hemispheric atrophy in the frontal regions (co-authors: MT, SC, and SK). Further, in agreement with the observation that stage 4 encephalitis occurs approximately 8 years after seizure onset and leads to hemispheric atrophy (Varghese et al., 2014), the brain regional volume in our patient, who had experienced seizures for 15 years, showed that the left anterior frontotemporal region was the most affected area (volume of atrophy/damage: 15.13 cm^3^, mean ± standard deviation: 248.05 ± 126.34). The volume of atrophy was estimated using OsiriX Lite (v.10.0.3, Switzerland) 3D medical image processing software.

Along with verifying of lateralized atrophy and functional lateralization (language and motor), details of laterality in the IGT-related neural circuitry in the patient were added as supplementary to the left-lateralized atrophy. Pre-surgical fMRI evaluations helped add further clarification in terms of the extent of lateralized atrophy in key brain areas identified to be critical to intertemporal IGT (Lin et al., 2008). Regional gray matter volume estimation of the ventrolateral PFC, dorsolateral PFC, orbitofrontal PFC, hippocampus, amygdala, thalamus, and anterior cingulate cortex was undertaken to add details related to lateral atrophy in the IGT circuitry. Gray matter analysis was performed by the second author (KC), using 3T MRI following the realignment, segmentation, and co-registration steps of SPM12 (Friston et al., 1995). Due to atrophy, the percentage of gray matter volume showed reduction in the left hemisphere. The regions in the left hemisphere with the most atrophy were the ventrolateral PFC (30%), dorsolateral PFC (10%), orbitofrontal PFC (10%), thalamus (23%) anterior cingulate cortex (21%), amygdala (14%), and the hippocampus (5%). Therefore, pre-surgery evaluation was to verify left-lateralized foci, and left-hemispheric atrophy in the patient and add information about the extent of laterality observed in the IGT-linked neural circuitry.

Recently, PFC lesion laterality study using a predominantly male-sample suggested that left hemisphere is critical for frequency-based attribute, specifically for infrequent punishment deck choices of deck D in the IGT decision making (Besnard et al., 2015). It was expected that the present case will enable us to explore the extent to which left-hemispheric atrophy and left-hemispheric dissociation will influence, attribute and phase-specific IGT decision making immediately (within 1 month) and after 1 year of hemispheric dissociation. Pre and post-surgery changes in the IGT decision were expected to be: (a) attribute specific, that is, differ for the cognition-intensive intertemporal decision making and for the emotion-focused, frequency-based decision-making, specifically for infrequent punishment deck B, and (b) phase-specific, that is, linked with uncertainty phase, due to its valence-directed anticipatory nature, and (c) linked with changes in valence-specific affect processing.

### Functional Specialization for Language

The presurgical fMRI evaluation was examined to ascertain the functional cerebral specialization for language for two reasons: (a) motor-related lateralization (right-handedness) affects decision making in the IGT in women (Singh, 2016), and seizure onset and right-side hemiparesis led to a shift from the patient’s original right-handedness to left-handedness. Since the conventional measure of motor specialization was inconsistent/ambiguous, functional specialization for language performed at the pre-surgical evaluation was examined. (b) Patients with epilepsy show atypical, i.e., right- rather than left-hemispheric, language specialization (Rausch and Walsh, 1984), and language-related variations affect post-surgical outcomes (Chaudhary et al., 2017). The fMRI language tasks consisted of word generation, which involved reading simple Hindi words (production), and comprehension, which involved correcting simple jumbled sentences (see Chaudhary et al., 2017 for task description and protocol for presurgical fMRI). Greater blood oxygen level-dependent activations were observed in posterior frontal and temporal regions, and the weighted-mean laterality index values for the frontal and temporal regions (0.56 and 0.6, respectively) suggested left-hemispheric lateralization of language (speech production and comprehension).

### Single-Case Design and the IGT

A pre-test–post-test design was used to assess preoperative and postoperative IGT decision making with a 3-day interval between the baseline test and surgery and a 30-day interval between surgery and the first retest, and the second retest after a 12 months gap from the surgery.

A day after the routine presurgical neuropsychological evaluation, the patient and her caretaker (mother) were presented an information sheet describing the study and procedures. Informed consent, which included information about preoperative evaluations (imaging, seismological, and neuropsychological evaluations) and presurgical and postsurgical assessments of the three tasks, was obtained from both the patient and the caretaker. The three tasks included were the International Affective Picture System (30 stimuli), Affect Norms for English Words (Hindi translated; 30 stimuli), and the IGT [100 trials of progressive reward versions (A′B′C′D′)]. With the aid of a research assistant, one experimenter (VS) administered the task to the patient in an outpatient department (OPD) room in the presence of the caretaker. The patient was comfortably seated on a bed with a backrest and a Hewett-Packard laptop (HP: ProBook) that was situated on a table in front of the patient to present the tasks. The first two tasks were <20 min each, and the patient was able to take a 5–7 min break between the first two tasks. Following the second task, the patient was given a 30 min break before starting the IGT (computerized IGT, PAR, FL, United States), which was administered using standardized bilingual (English and mother tongue) task instructions. A research assistant served as a proxy to mark the patient’s selections on the computer because the patient demonstrated weakness in the right hand/limb, unfamiliarity with operating a computer, and brief seizures during the tasks. Importantly, after the completion of each seizure, the patient was enabled to continue or discontinue the tasks. The total duration of the experimental session was 2 h; the IGT lasted for 24.09 min. Five seizures that each lasted <2 min occurred during the IGT (trials 11–12, 24–25, 34–35, 66–67, and 87–88). When asked if she wanted to continue/discontinue, the patient always preferred to continue and appeared to immediately resume interest in the tasks. The patient may have been more motivated to complete tasks than the preoperative neuropsychological evaluation because they latter were presented on a computer: the three digital tasks involved a livelier response format that was more entertaining for the patient (all three tasks involved “smileys” as the response format; smileys indicated positive valence in the picture and word tasks and the amount won in the IGT), while the neuropsychological assessment was paper-and-pencil based and non-interactive.

The two postoperative reassessments investigated the effects of disconnecting the epileptogenic left hemisphere. At 1 month’s post-surgery assessment (follow up 1), the patient was seizure free, exhibited slight swelling in the right foot, and could move her right hand with greater facility, at the 1-year post surgery assessment (follow up 2), the patient was seizure free, and there was no leg swelling. The same protocols that were used at baseline testing were repeated during the two follow up reassessments, emotion pictures were followed by words, followed by the IGT. The patient’s speech did not evince any noticeable changes after surgery. At the first follow-up, patient had no recollection of the IGT task, but we observed moments of insight as the task progressed – e.g., exclamations of “ah-ha” in between trials – however, these moments were transitory. Her interest in the task appeared to be the same as that demonstrated when she performed it the first time. At the second follow-up, the patient had recollection of performing the IGT, but continued to explore the task showing no visible/explicit effects of being familiar with the task.

### The IGT Attributes (Intertemporal and Frequency)

The IGT task features four decks labeled A′, B′, C′, and D′, and this progressive reward version was purchased from the Psychological Assessment Resources (PAR, FL, United States). It uses the same reward–punishment structure as used in the original task, except that the difference between the intertemporally advantageous and disadvantageous decks is more prominent/evident (Bechara et al., 2000). As per the intertemporal attribute, decks A′ and B′ are risky because they are poor long-term choices that yield high magnitude long term punishment, whereas decks C′ and D′ are safely profitable in the long term because of low magnitude and few long term punishment. Frequency-based attribute suggests that decks A′ and C′ are risky because of frequent punishment, and decks B′ and D′ are preferred due to infrequent punishment, lowest frequency of punishment being in the deck B′. Deck choices at baseline and retests were calculated based on two attributes: (1) intertemporal (decks A′ and B′ vs. decks C′ and D′) and (2) frequency-based choices (decks B′ and D′ vs. decks A′ and C′). The deck analysis of the patient’s preoperative and postoperative choices was performed for the first and last halves of the task trials to assess decision making under uncertainty and risk, respectively (Steingroever et al., 2013).

Mixed analyses of variance (ANOVAs) were conducted using SPSS version 21 to analyze the presurgical and postsurgical deck choices at follow-up 1 and 2 (intertemporal: decks A′ and B′ and decks C′ and D′; frequency: decks B′ and D′ and decks A′ and C′) for 100 trials (1 block = 20 trials, 5 blocks, as per Bechara et al., 2005) and for the 100 trials that were divided into two trial phases (trials 1–50 = uncertainty block and trials 51–100 = risk block, as per Steingroever et al., 2013). The deck choice was the within-subject variable and block was the between-subject variable [5 blocks in case of 100 trials task progression and 2 blocks in case uncertainty (trials 1–50) and risk phase (trials 51–100)]. The level of statistical significance was set at *p* < 0.05.

### Affect and Valence Processing

International Affective Picture System [two sets of 30 stimuli (set 1 and set 2), 10 stimuli for each of the three valence: positive, negative, neutral] (Lang et al., 1999), set 1 was used at presurgery, and set 2 at follow-up 1, both sets were used at follow up 2 (set 2 followed by set 1). Affect Norms for English Words (Hindi translated; 30 stimuli, 10 stimuli for each of the three valence: positive, negative, neutral) (Bradley and Lang, 1999) were used to assess valence processing at three assessments. Picture stimuli were presented on computer screen one by one in the order of 10 positive, 10 negative, and 10 neutral pictures, whereas the words were read out (by the first author, VS) one by one in two blocks each block of 15 words (positive words followed by neutral words, and negative words). Valence ratings from the database of the stimuli were as follows (negative stimuli mean valence rating = 1–3, neutral stimuli mean valence rating = 4–6, and positive stimuli mean valence rating = 7–9); all arousal ratings were above 2. Patient was asked to respond to the picture in term of how it made her feel by pointing to one of the three smiley faces each depicting a valence of negative/frown, and neutral/normal face, and positive/smile (0 = negative, 1 = neutral, 2 = positive), with the spontaneity with which the patient responded being assessed on a 3-point scale (1 = very hesitant and unclear while responding, 2 = somewhat hesitant and unsure while responding, 3 = immediate and confident while responding). Ratings were calculated by considering two attributes, the valence reported and the spontaneity of valence response (1 = low, 2 = medium, and 3 = high) to produce ratings, for instance a picture rated as ‘0’ or negative, and with immediate and confident responding ‘3’ will produce a rating of ‘0 + 3 = 3.’ This procedure enabled simplification of valence processing assessment (unlike the elicitation of affect response on a 9-point rating scale) as well as enabling a comparison of the obtained response with the 9-point response scale that is used in affect database to assess valence (i.e., low ratings indicate negative valence, mid-scale ratings indicate neutral valence, and high ratings indicate positive valence). Aligned with the valence-specific hemispheric lateralization (Davidson, 1992), it was expected that post-surgery affect response/ratings (pictures and words) will show valence-specific alteration. Mixed analyses of variance (ANOVAs) were conducted using patient’s stimuli ratings as within-subject variable (pre-surgery vs. post-surgery at follow up assessment 1 vs. follow up assessment 2) and valence (negative vs. neutral vs. positive) as between-subject variable. Analysis addressed pictures and words separately; and pictures at follow-up 2 used the ratings of set 1 (pictures shown prior to surgery) and of set 2 (pictures shown at post surgery follow up 1), in other words presurgery ratings (set 1), post surgery follow up 1 ratings (set 2), and post surgery follow up 2 ratings of set 1, and post surgery follow up 2 ratings of set 2 served as four levels of within-subject variable for analysis of valence processing.

## Results

We first examined IGT deck choices across the 100 trials of the task to determine whether choice of decks alters with task progression, separately for the two attributes (intertemporal: decks A′ and B′ vs. decks C′ and D′; frequency-based: decks B′ and D′ vs. decks A′ and C′) assessed at three points (pre- and post-surgery assessments).

Specifically, in terms of attribute and task progression across the five blocks of trials, our analysis revealed a main effect of intertemporal deck choice (decks A′ and B′ vs. decks C′ and D′) [*F*(1,95) = 4.38, *p* = 0.04] and of deck × block interaction [*F*(4,95) = 2.52, *p* = 0.05] on presurgical performance, disadvantageous deck choices were prominent, and they altered as the trials progressed. There was no main effect of decks, but a significant deck × block interaction was observed on the first post-surgical performance within 30 days of surgery [*F*(4,95) = 2.79, *p* = 0.03], whereby disadvantageous deck choices altered only as the task progressed. After 1 year at the second postsurgical follow up, there was a main effect of intertemporal deck choice [*F*(1,95) = 23.70, *p* < 0.001] but the deck × block interaction was non-significant suggesting choices from the disadvantageous decks increased, independent of the task progression. Unlike presurgical behavior, differentiation of long-term advantageous decks vs. short-term disadvantageous decks seemed contingent on task trials at the first postsurgical follow up within 30 days of the surgery, but reverted to the presurgery trend, that is, prominent preference for disadvantageous decks, independent of task progression.

**FIGURE 2:**
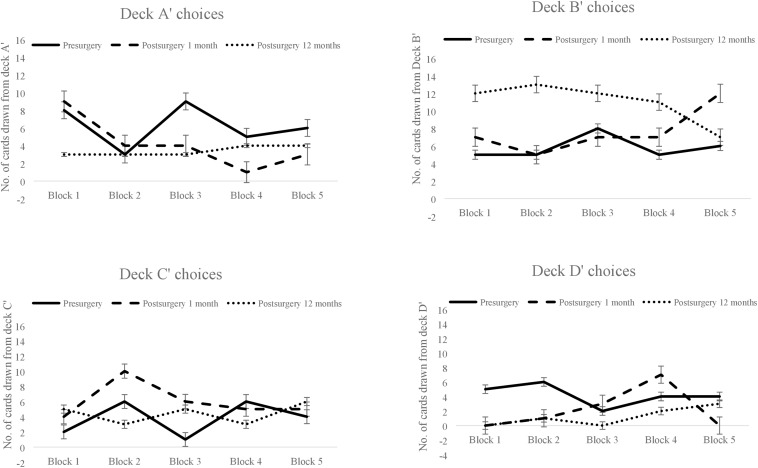
The patient’s pre- and post-surgical block-wise choices for each of the four decks in the Iowa Gambling Task. Block-wise deck A′ choices made pre-surgery, post-surgery within 1 month, and post-surgery after 12 months (top left-hand corner), similarly deck B′ choices (top right-hand corner), deck C′ choices (bottom left-hand corner) and deck D′ choices (bottom right-hand corner). Error bars show the standard error.

There was no main effect of frequency-based deck choice (decks B′ and D′ vs. decks A′ and C′) [*F*(1,95) = 0.00, *p* = 1.00] or of a deck × block interaction [*F*(1,95) = 0.09, *p* = 0.98] on pre- and post-surgical follow up performance within 1 month. One year of post-surgery follow up suggested main effect of deck choices, there was a prominent preference for infrequent punishment decks B′ and D′ [*F*(1,95) = 6.24, *p* = 0.01]. Frequency-based preference was undifferentiated and unaffected by task trials at presurgery, and remained undifferentiated within a month of surgery but showed alteration after 1 year (see Figure 2).

**FIGURE 3:**
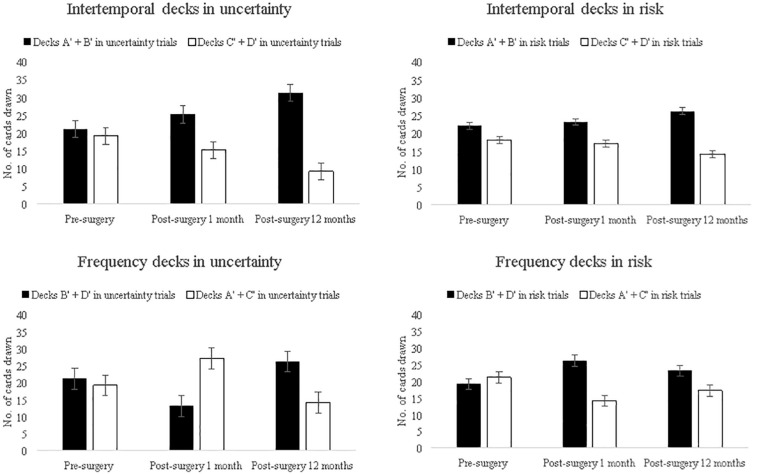
Number of cards drawn from long-term risky decks (A′ + B′) and safe decks (C′ + D′) during uncertainty (top left-hand corner) and risk trials (top right-hand corner) before and after surgery, and number of cards drawn from infrequent punishment decks (B′ + D′) and frequent punishment decks (A′ + C′) during uncertainty (bottom left-hand corner) and risk trials (bottom right-hand corner) before and after surgery. Error bars show the standard error.

Next, we examined if deck choices in uncertainty and risk trials vary pre and post-surgery follow-ups. To test if the two attributes were differentially sensitive to the uncertainty- and risk-related trials/phases of IGT decision making, changes in intertemporal deck choices at three assessments (pre-surgery vs. post-surgery follow up 1 vs. follow up 2) (e.g., decks A′ and B′ at pre- and post-surgery follow up at 1 month and at 12 months) were analyzed as within-subject variable and the task phase (uncertainty and risk) as the between-subject variable. Four separate analyses were performed, two for the two intertemporal deck choices (decks A′ and B′, and decks C′ and D′) and two for the two frequency-based choices (decks B′ and D′, and decks A′ and C′). There was no main effect of deck [*F*(2,196) = 2.36, *p* = 0.10] or a block × deck interaction for decks A′ and B′ [*F*(2,196) = 0.41, *p* = 0.68]. Similarly, neither a main effect of deck nor a deck × block interaction was identified for decks C′ and D′ suggesting that intertemporal choice decks of decks A′ and B′ and C′ and D′ under uncertainty and risk across surgery and the two follow up assessments remained constant. There was no main effect of frequent punishment decks, but a significant deck × block interaction for decks A′ and C′ was observed at 1 month follow up which demonstrated a postsurgical increase in the selection of frequent punishment decks (decks A′ and C′) under uncertainty but a decrease under risk [*F*(1,98) = 14.33, *p* < 0.001], which reversed after 1 year, that is, choices from frequent punishment decks decreased under uncertainty but increased under risk. For decks B′ and D′, there was a main effect of deck [*F*(2,196) = 3.03, *p* = 0.05] suggesting that compared to pre-surgery, fewer cards were drawn from these decks at the 1 month follow up, but infrequent punishment deck choices increased after 1 year of surgery. The deck × block interaction [*F*(2,196) = 8.33, *p* < 0.001] was significant, indicating a postsurgical reduction in the choice of infrequent punishment decks (decks B′ and D′) under uncertainty but an increased selection of the infrequent punishment decks under risk phase at 1-month follow up. After a year, the reverse was observed, that is, choices from infrequent punishment decks increased under uncertainty and decreased under risk (see Figure 3).

**FIGURE 4:**
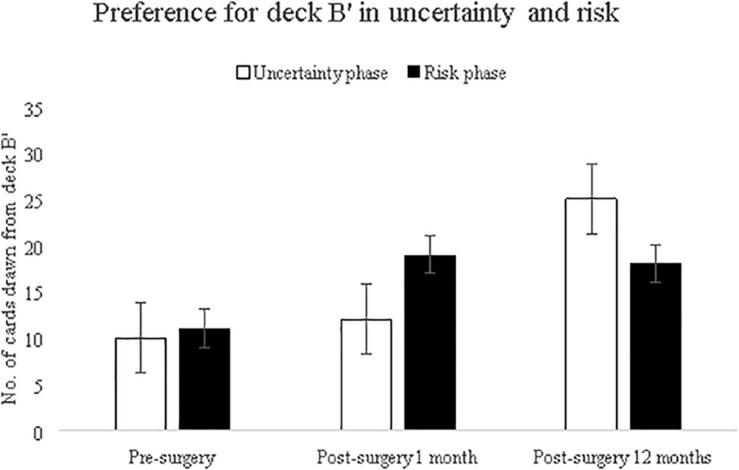
Number of cards drawn from infrequent punishment deck B′ during uncertainty and risk trials before and after surgery. Error bars show the standard error.

Next, we examined whether choice of deck B varies with task progression, and varies across the uncertainty-risk phase. To investigate frequency-based attribute, specifically the ‘Deck B’ preference that is observed widely in female participants (Overman and Pierce, 2013), we analyzed deck B choices (before surgery, immediately after 1 month, and after 12 months) using two separate analysis, one addressing deck B choice and the task progression (five blocks), and second addressing selection of deck B choices across two phases (uncertainty and risk). Results for task progression showed main effect of deck B [*F*(2,190) = 7.66, *p* = 0.001] suggesting that number of cards drawn from deck B increased across the three assessments; non-significant interaction of deck × blocks suggests that task progression did not contribute to this increase. Results for uncertainty and risk phase showed main effect of deck B [*F*(2,196) = 7.72, *p* = 0.001], and deck × blocks interaction was significant [*F*(2,196) = 3.50, *p* = 0.03] suggested that the deck B choices varied across the three assessments in the uncertainty phase (see Figure 4).

**FIGURE 5:**
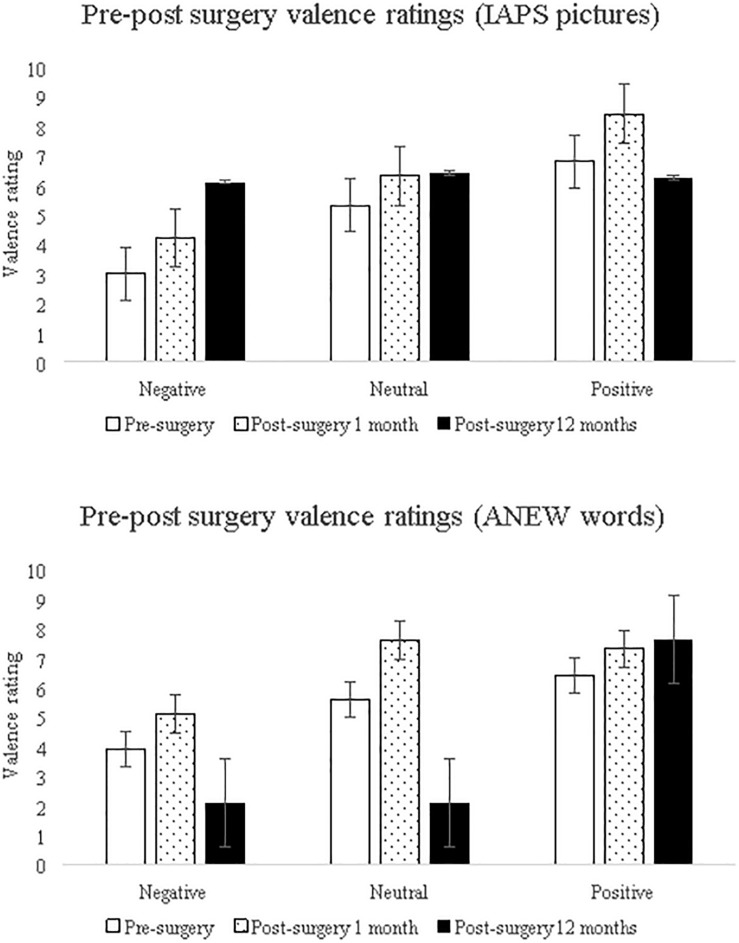
The patient’s valence-specific pre- and post-surgical affect ratings using affect-eliciting pictures (IAPS: top) and words (ANEW: bottom). Error bars show the standard error.

Lastly, valence processing was examined using affect pictures and results showed main effect for affect ratings [*F*(2,54) = 4.57, *p* = 0.02] suggesting that affect processing altered from pre- to post-surgery follow ups, and the interaction of affect ratings and valence was significant [*F*(4,54) = 3.80, *p* = 0.01] suggesting that the affect alteration was valence-specific. Valence processing was examined using affect words and the results showed main effect of affect ratings [*F*(2,54) = 24.09, *p* < 0.001] suggesting alteration of affect processing of words across pre- and post-surgery follow ups as well as the interaction of affect ratings and valence was found to be significant [*F*(4,54) = 10.35, *p* < 0.001] suggesting valence-specific alteration in word affect processing (see Figure 5). Using two types of affect eliciting stimuli (pictures and words), the results suggests that affect processing altered across pre- to post-surgery assessments, and that valence contributed to this alteration in affect processing.

## Discussion

We first used pre-surgical neuroimaging evaluations of a patient with unilateral brain damage (left-hemispheric Rasmussen encephalitis) to ascertain (a) uni-hemispheric atrophy, (b) functional lateralization of language functions, and (c) lateralized atrophy in neural circuitry crucial to the performance of the IGT. Next, we examined cognition-intensive intertemporal and emotion-based frequency-based processing of deck choices by comparing the patient’s preoperative and postoperative performances on the IGT at three assessment points: pre-surgery and post-surgery (after 30 days and after 12 months from the surgery). Additionally, we examined valence-specificity in pre- and post-surgery alteration of affect processing.

Our results showed that prior to hemispheric dissociation surgery, the patient made more disadvantageous choices, and this preference changed as the trials progressed. Within 30 days of the surgical dissociation at follow-up 1, drawing of risky decks progressed only as the task progressed, but after 1 year, more disadvantageous deck choices were observed and this preference for disadvantageous decks remained unaffected by task progression. The patient showed indifference to frequency-based deck choices prior to the surgery, and this indifference continued after 30 days of the surgery, however, frequency-based deck choice, specifically preference for infrequent punishment decks was observed after 1 year of surgery, and this preference was independent of task progression. Thus, results of the two attributes in a case of uni-hemispheric left atrophy, and pre and post-hemispheric dissociation offered an opportunity to examine the two attributes: cognition-intensive intertemporal-based decisions showed alteration in terms of its dependency on task progression at the three assessment points, whereas task-progression did not contribute to frequency-based decision making either pre- or at post-surgery.

On comparing the extent to which the two attributes respond to uncertainty and the risk phase of the IGT task, results suggests that the intertemporal choice decks (long term advantageous decks C and D, as well as disadvantageous decks A and B) in uncertainty and risk phase of the task remained un-altered after surgery, whereas the frequency-based choice decks (infrequent punishment decks B and D as well as frequent punishment decks A and C) in uncertainty and risk phase showed pre- and post-surgery alteration. As expected, phase-specificity of frequency-based choices altered pre- and post-surgery, most notably, the choice of infrequent punishment deck B in the uncertainty phase varied from pre- to post-surgery assessment. Thus, after the hemispheric-dissociation surgery, frequency-based preferences reversed in uncertainty and risk trials, and as expected, were phase-specific. These findings align with those of Besnard et al. (2015), who observed that the frequency-based choices of patients with frontal lobe damage and healthy participants differed in uncertainty trials but not in risk trials. Compared to intertemporality-based selection, frequency-based decision making may be differentially sensitive to uncertainty and risk in the IGT, and hemispheric specialization might be a potential contributor to this phase-sensitivity. Studies documenting effects of laterality of atrophy should consider both the attributes of decision making, as well as include phase-specificity into consideration because it is possible that frequency-based decision making alteration may be specific to the uncertainty vs. risk phase of the IGT, leaving intertemporal decision making unaltered across the two phases.

Together with existing data, the present findings add support to the hemispheric involvement in the IGT decision making (Clark et al., 2003; Tranel et al., 2005). For instance, lateralized sex-differences, that is male-advantage in intertemporal choices is attributed to right hemisphere (van den Bos et al., 2013) whereas the female disadvantage is attributed to usage of frequency-based processing and is linked with left-laterality (Lin et al., 2007; Overman and Pierce, 2013), the present result expand this sex-specific hemispheric role and are suggestive of further attribute, and phase-specificity in the IGT. According to the hierarchical information processing theory, the right hemisphere specializes in the processing of global features, whereas the left hemisphere specializes in local processing (Fink et al., 1996). Studies have reported that frequency-based IGT choices reflect automatic processing (Wilder et al., 1998; Stocco et al., 2009); specifically the selection of the infrequent punishment deck B recruits the left hemisphere (Lin et al., 2008). Additionally, the right hemisphere specializes in cognitive control in uncertainty (Garavan et al., 1999; Goel et al., 2007); therefore, it is possible that the intertemporal decision making in the IGT reflects right-lateralized, global processing, whereas the emotion-based automatic and local processing of frequency is left-lateralized, and probably it is the latter that is differentially sensitive to uncertainty and risk in the IGT. Therefore, the present results lend support to hemispheric specialization in the IGT intertemporal decision making (Clark et al., 2003; Tranel et al., 2005), and further draw attention to attribute, and phase-specificity potentially impacting decision making in the IGT decision making.

It was further speculated that attribute, and phase-specific alteration observed post-hemispherotomy might be linked to valence processing. As expected, affect processing (pictures and words) showed valence-specific alteration suggesting that processing of valence (negative, neutral, and positive) was altered from pre-surgery to the two assessments carried out within 1-month and 12-month post-surgery. In line with the valence hypothesis, that is, left-lateralized positive valence processing and right-hemispheric specialization for negative valence (Davidson, 1992), post-hemispheric dissociation showed valence-specific alteration in affect processing. A recent study found that in comparison to controls, epilepsy patients showed increased affect ratings to affect pictures (IAPS), and ratings were independent of valence, however, the study recruited mixed-gender epilepsy patients (males > females) with mixed laterality (right-side patients > left) (Ciuffini et al., 2018). It is possible that altered valence-processing contributes to disadvantageous decision making in uni-lateral epilepsy patients, however, the effect of laterality in affect processing itself remains poorly understood (Hixson and Kirsch, 2009). Recently, processing of affect words (ANEW) showed valence-specific laterality in normal healthy participants (Martin and Altarriba, 2017). The implication of altered valence-processing linked with IGT decision making implies that the preference for the infrequent punishment deck B that is widely observed in females might be linked to valence-related lateralization, and potentially contributing to sex differences in attribute and phase-specificity in the IGT (i.e., emotion-based frequency attribute sensitive to uncertainty phase in females). Within the context of epilepsy, localization of language observed prior to seizure onset, during seizure, and post-seizure (i.e., pre, ictal, post-ictal) has been used for predicting epilepsy surgery outcomes. It is possible that affect-linked valence-processing and affect-guided decision making such as that observed in the IGT, could potentially serve as a lateralized constructs that can be useful in predicting the extent of decision-making deficits and quality of life outcomes in the epilepsy patients.

There were several limitations of the current study. As recommended (Fisch, 2001), single-case designs utilized visual inspections of data in combination with pre- and post-test comparisons, a larger gender-balanced sample of patients with uni-hemispheric atrophy are needed to fully examine hemispheric specialization in attribute (intertemporal vs. frequency-based) and phase-specific (uncertainty vs. risk) IGT decision making. Neuropsychological evaluations of frontal lobe-based functions (i.e., executive functions, esp. working memory) may have facilitated the interpretation of our results; however (a) the patient’s incomplete neuropsychological evaluations indicate the presence of cognitive impairment, which is a hallmark of drug-refractory Rasmussen encephalitic epilepsy (b) the patient’s emotion/affect processing, and affect-aided decision making that is independent of cognitive resources (i.e., independent of cognitive impairment), is the focus of this investigation. Similarly, the current study did not evaluate mood, which could have impacted performance. Future studies should employ neuroimaging during the IGT to identify the neural circuitry recruited by patients with lateralized hemispheric damage both before and after surgery. We expect that this first IGT study of pre and post-hemispheric dissociation will give way to future studies that will identify potential cerebral specialization in the IGT decision making, after accounting for executive functions such as working memory resources.

## Conclusion

Evaluating decision making in a female patient with uni-hemispheric atrophy in the language-specialized left-hemisphere across hemispheric disconnection, this single-case study improves our understanding of the potential hemispheric specialization for intertemporal- and frequency-based decision making during two phases of the IGT (uncertainty and risk). Sex differences in the IGT have been attributed to greater right lateralization of intertemporal decision making in men, and left lateralization and preference for infrequent punishment decks in females (Tranel et al., 2005; van den Bos et al., 2013). Preoperative preference for infrequent punishment decks was absent in this female patient with left hemispheric atrophy, but we observed this preference to increase post-surgery, and we further observed that this preference is prominent in the uncertainty phase of the IGT, possibly due to uncertainty phase characterized by valence. Even though our results contribute to the understanding of right-lateralization and sex differences in the IGT, male–female comparisons between the IGT performance done before and after hemispheric dissociation are needed to test this assumption.

## Data Availability Statement

The datasets generated for this study are available on request to the corresponding author.

## Ethics Statement

Written informed consent was obtained from the individual for the publication of any potentially identifiable images or data included in this article.

## Author Contributions

VS conceived and carried out the study. KC and SK carried out the brain imaging and analysis. MT and SC performed the diagnosis, surgery/treatment and planned the follow ups. All authors had equal contribution.

## Conflict of Interest

The authors declare that the research was conducted in the absence of any commercial or financial relationships that could be construed as a potential conflict of interest.
